# The landscape of human genes involved in the immune response to parasitic worms

**DOI:** 10.1186/1471-2148-10-264

**Published:** 2010-08-31

**Authors:** Matteo Fumagalli, Uberto Pozzoli, Rachele Cagliani, Giacomo P Comi, Nereo Bresolin, Mario Clerici, Manuela Sironi

**Affiliations:** 1 Scientific Institute IRCCS E. Medea, Bioinformatic Lab, Via don L. Monza 20, 23842 Bosisio Parini (LC), Italy; 2 Bioengineering Department, Politecnico di Milano, P.zza L. da Vinci, 32, 20133 Milan, Italy; 3 Dino Ferrari Centre, Department of Neurological Sciences, University of Milan, IRCCS Ospedale Maggiore Policlinico, Mangiagalli and Regina Elena Foundation, Via F. Sforza 35, 20100 Milan, Italy; 4 Department of Biomedical Sciences and Technologies LITA Segrate, University of Milan, Via F.lli Cervi 93, 20090 Milan, Italy; 5 Don C. Gnocchi ONLUS Foundation IRCCS, Via Capecelatro 66, 20148 Milan, Italy

## Abstract

**Background:**

More than 2 billion individuals worldwide suffer from helminth infections. The highest parasite burdens occur in children and helminth infection during pregnancy is a risk factor for preterm delivery and reduced birth weight. Therefore, helminth infections can be regarded as a strong selective pressure.

**Results:**

Here we propose that candidate susceptibility genes for parasitic worm infections can be identified by searching for SNPs that display a strong correlation with the diversity of helminth species/genera transmitted in different geographic areas. By a genome-wide search we identified 3478 variants that correlate with helminth diversity. These SNPs map to 810 distinct human genes including loci involved in regulatory T cell function and in macrophage activation, as well as leukocyte integrins and co-inhibitory molecules. Analysis of functional relationships among these genes identified complex interaction networks centred around Th2 cytokines. Finally, several genes carrying candidate targets for helminth-driven selective pressure also harbour susceptibility alleles for asthma/allergy or are involved in airway hyper-responsiveness, therefore expanding the known parallelism between these conditions and parasitic infections.

**Conclusions:**

Our data provide a landscape of human genes that modulate susceptibility to helminths and indicate parasitic worms as one of the major selective forces in humans.

## Background

Helminth infections are estimated to infect about 2 billion individuals worldwide (reviewed in [1]). Although rarely fatal, these parasites cause high rates of morbidity by establishing chronic infections. In particular, the highest parasite burdens are observed in pre-school and school-aged children, often resulting in anemia, undernourishment and growth stunting (reviewed in [1]). During pregnancy helminth infection is a risk factor for preterm delivery, reduced birth weight and maternal mortality (reviewed in [1]). Moreover, by chronically infecting their host, parasitic worms increase the susceptibility to other pathogens such as viruses, bacteria and protozoa [1]. Previous works have indicated that the intensity of helminth infection is a heritable trait, although measures of heritability vary among studies and depend on the parasite beanalyzed [2].

These observations suggest that helminth infections have represented a very strong selective pressure for humans, a selective pressure that is very likely to also be remarkably constant over time. Indeed, most vertebrates have been hosting a wide range of parasitic worms for million years and humans have had their share before emerging as a species (reviewed in [3]). We have previously addressed the role of helminths as selective agents in human evolution analyzing a large set of human genes encoding interleukins and their receptors; we demonstrated that the pressure imposed by parasitic worms on these genes has been stronger than the one due to viral and microbial agents [4]. The reasons for this observation likely lie in the long-term relationship between humans and helminths, in the relatively slow evolutionary rates of these parasites and in their geographic distribution being considerably stable. Here we aimed at exploiting the selection signatures left by these pathogens on human genes to identify, at the genome-wide level, candidate genes and variants that may have been subjected to helminth-driven selective pressure.

## Results

### Helminth diversity and prevalence correlate across geographic locations

In order to search for candidate variants subjected to helminth-driven selection, an estimate of the selective pressure exerted by these pathogens needs to be defined. It has been previously suggested [4-6] that the selective pressure exerted by infectious diseases in different geographic areas can be estimated by counting the number of different pathogen species/genera that are transmitted in these regions. In particular, in the case of helminthiases, we consider parasite diversity to be a better estimate of helminth-driven selective pressure than prevalence for different reasons. First of all, comprehensive data on prevalence are impossible to retrieve for many parasite species/genera or countries and even when available, prevalence data may vary considerably within the same country depending on the surveyed regions and the population surveyed (e.g. city dwellers rather than farmers/bushmen/nomads, children rather than adults). Moreover, the prevalence of specific helminthiases might have changed greatly over recent years as a result of eradication campaigns, and historical prevalence data are rarely available. Also, it should be considered that prevalence data are difficult to combine since in endemic regions polyparasitism is common [1]; indeed, in these regions subjects infected with multiple helminths tend to harbor the most intense infections, possibly due to an additive and/or multiplicative impact on nutrition and organ pathology (reviewed in [7]).

These same observations also apply to other possible measures such as parasite burden or infection pathogenicity, which are very difficult to quantify and that may have varied considerably along human evolutionary history. Conversely, the diversity of helminth species transmitted in different geographic locations has been shown to depend upon climatic variables [8] which, in turn, may be considered as relatively stable over time, suggesting that diversity may better describe long-term evolutionary pressures. Thus, we calculated helminth diversity from the Global Infectious Disease and Epidemiology Network database (Gideon); as described in the method section, all entries for single helminthiases were manually inspected and all species/genera transmitted in a given location were counted as present irrespective of their prevalence. The number of different helminth species/genera per country is reported in Additional file 1 (Table S1).

We next wished to verify whether the prevalence of the most common helminth infections (as reported in [1]) correlates with helminth diversity across the 52 populations genotyped in the HGDP-CEPH panel (Additional file 1, Table S1). Again, we retrieved prevalence and diversity data from Gideon by manually inspecting single and retrieving all prevalence surveys. The prevalence of single helminthiases per country was obtained by averaging all surveys; data obtained on HIV-seropositive individuals were not included in the analyses because of the known modulation of HIV on the susceptibility on helminth-based infections [1,9,10]. Finally, countries with no survey data were not included in the analysis for the specific helminth. Parasites were then grouped into five major classes (following [1]). As shown in table 1, the prevalence of all parasite groups correlated with helminth diversity.

**Table 1:** Correlation between the prevalence of all parasite groups and helminth diversity

Parasite group	Kendall's τ	*p *value	Parasite species
Soil-transmitted nematodes	0.381	0.00037	*Ascaris lumbricoides, Trichuris trichiura, Necator americanus, Ancylostoma duodenale*
Filarial nematodes	0.387	0.00053	*Wuchereria bancrofti, Brugia malayi, Onchocerca volvulus, Loa loa^a^*
Schistosomes	0.482	2.8 × 10^-5^	*Schistosoma mansoni, Schistosoma haematobium, Schistosoma intercalatum, Schistosoma japonicum, Schistosoma mekongi*.
Food-borne trematodes	0.617	2.6 × 10^-7^	*Clonorchis sinensis, Opisthorchis viverrini, Paragonimus africanus, Paragonimus compactus, Paragonimus ecuadoriensis, Paragonimus hueitungensis, Paragonimus heterotremus, Paragonimus kellicotti, Paragonimus mexicanus, Paragonimus miyazakii, Paragonimus szechuanensis, Paragonimus tuanshanensis, Paragonimus uterobilateralis, Paragonimus westermani, Fasciolopsis buski, Fascicola (hepatica or gigantica)*
Taenia^b^	0.462	0.00633	*Taenia solium*

a *Dracunculus medinenis *was not included because prevalence data were only available in few countries;

b Prevalence data were available for 27 populations only.

### SNPs associated with helminth diversity are over-represented in immune response genes

Previous analysis have indicated that several genes coding for interleukins and interleukin receptors are subjected to helminth-driven selective pressure [4]. Interleukins are central mediators of immunity and inflammation and, in general, we might expect genes with a role in immune response to be preferential targets of helminth-driven selective pressure. Specifically, we expect variants within these genes to be more frequently associated with helminth diversity than observed for randomly sampled loci. We verified this prediction by analyzing the ImmPort list which contains 2,287 genes involved in immune response and covered in the HGDP-CEPH panel, this latter containing data for more than 660,000 single nucleotide polymorphisms genotyped in almost 950 individuals sampled throughout the world (Additional file 1, Table S1) [11].

We calculated Kendall's τ rank correlation coefficient between allele frequencies of SNPs in ImmPort genes and helminth diversity. A SNP was defined as being significantly associated with helminth-diversity if it displayed a significant correlation (*p *value after Bonferroni correction < 0.01; uncorrected *p *value < 1.94 × 10^-7^) and a τ value higher than the 95th percentile in the distribution of correlation coefficients calculated over all SNPs having minor allele frequency (MAF) similar (in the 1% range) to that of the SNP being analyzed. This latter requirement stems from the need to account for the influence of non-selective events, as a few HGDP-CEPH population result from recent or ancient admixture [11] and population history, migratory events and genetic drift have affected human genetic variability [12,13]. Among 2,287 ImmPort genes, 246 contained at least one SNP significantly associated with helminth diversity (Additional file 2, Table S2). The likelihood to obtain an equal or higher number of genes carrying significantly associated SNPs was assessed by a re-sampling approach. Specifically, we divided all genes with at least one SNP typed in the HGDP-CEPH panel in 24 intervals based on the number of typed SNPs; 10,000 re-samplings were then performed by selecting for each ImmPort gene, a randomly selected gene with a similar number of SNPs (i.e. a gene located in the same interval) (see methods). The number of SNPs in ImmPort genes did not differ significantly from the average number in the re-samplings (p = 0.22) and the empirical probability of obtaining 246 genes with at least one significant SNP resulted equal to 0.041, indicating that immune response genes more frequently display variants correlating with helminth diversity compared to randomly chosen loci.

When the same analysis was performed using the prevalence of soil-transmitted nematodes, filarial nematodes, schistosomes and food-borne trematodes no significant enrichment for ImmPort genes was observed (empirical *p *values = 0.83, 0.96, 0.45, and 0.39, respectively).

In agreement with previous suggestions [4], these data indicate that helminth diversity may be a reliable estimator helminth-driven selective pressure.

### Genome-wide search for variants subjected to helminth-driven selective pressure

Given these results, we wished to identify SNPs significantly associated with helminth diversity on a genome-wide base. We therefore calculated Kendall's rank correlations between allele frequency and helminth diversity for all SNPs (n = 660,832) typed in the HGDP-CEPH panel. We next searched for instances which withstood Bonferroni correction with α = 0.05 (i.e. uncorrected *p *value < 7.6 × 10^-8^) and displayed a τ percentile rank higher than the 95th among MAF-matched SNPs. A total of 3,478 SNPs mapping to 810 distinct genes satisfied both requirements (Additional file 3, Table S3). We next verified whether climatic variables could be responsible for the correlations detected between these SNPs and helminth diversity. Hence, for all countries where HGDP-CEPH populations are located we obtained the following parameters: average annual minimum and maximum temperature, short wave radiation flux and precipitation rate (annual maximum and mean). None of these SNPs withstood Bonferroni corrections in these analyses.

Previous works have reported an enrichment of selection signatures within or in close proximity to human genes [12,14-17]. In line with these data we verified that helminth-associated SNPs are more frequently located within gene regions compared to a control set of MAF-matched variants (χ^2 ^test, *p *= 0.014).

A full list of the 3,478 SNPs that showed a significant correlation with helminth diversity is available as Additional file 3 (Table S3). Table 2 shows the 20 strongest correlations together with a short comment on the possible role of candidate genes in immune response or helminth resistance.

**Table 2:** Top 20 SNP (or SNP clusters) associated with helminth diversity.

SNP	Candidate gene	Distance (bp)^a^	Annotation	τ	Description	Reference
rs6989916	*CSMD1*	14833	intergenic	0.702	CSMD1 acts as a regulator of the complement system	[77]
rs11614925; rs2082529	*NAP1L1, PHLDA1*	35490; 1923	intron; intergenic	0.700; 0.700	PHLDA1 participates in regulating T-cell receptor/CD3-dependent induction of CD95/Fas	[78]
rs1369977; rs1369976	*PDHA2*	45648; 44682	intergenic	0.696; 0.687	Pyruvate dehydrogenase (lipoamide) alpha 2	
rs4684083; rs9681213; rs1516320	*CHL1*	49940; 175000; 162760	intergenic	0.690; 0.676; 0.675	Cell adhesion molecule with homology to L1CAM	
rs10014145	*SLC39A8*	genic	intron	0.685	*SLC39A8 *encodes a zinc transporter which is up-regulated by different cytokines in lung epithelia and monocytes	[79,80]
rs4682429	*CD200R1L*	12582	intergenic	0.684	Engagement of CD200RL1 (aka CD200R2) results in the development of dendritic cells that preferentially induce Treg Cells	[33]
rs7130880	*PRMT3*	14431	intergenic	0.682	*PRMT3 *encodes a protein arginine methyltransferase expressed in T and B cells; arginine methylation is important for T cell activation and is induced by CD28 engagements	[81]
rs504508	*KATNAL1*	111767	intergenic	0.682	Katanin p60 subunit A-like 1	
rs12371626	*GRIP1*	genic	intron	0.680	GRIP1 acts with Beta-catenin to enhance the activity of LEF1 (lymphoid enhancer-binding factor 1)	[82]
rs1441443	*PDZRN3*	252500	intergenic	0.678	PDZ domain containing ring finger 3	
rs236233	*IRAK1BP1*	452153	intergenic	0.677	IRAKBP1 is required for TNF-alpha activation of NF-kB dependent-gene expression	[83]
rs3807250	*DPP6*	genic	intron	0.677	Dipeptidyl-peptidase 6; a susceptibility gene for amyotrophic lateral sclerosis	[84]
rs11702528	*BTG3*	52819	intergenic	0.676	*BTG3 *is a transcriptional target of p53 that inhibits E2F1	
rs985122	*AJ606331*	508	intergenic	0.674	Putative non-coding RNA	
rs4692241	*STIM2*	529000	intergenic, eQTL	0.673	STIM2 promotes store-operated Ca2^+ ^entry into T cells; STIM1 and STIM2 proteins are required for the development and function of regulatory T cells	[34]
rs10270302	*GIMAP7*	genic	intron	0.672	*GIMAP7 *encodes a GTPase of the immunity-associated protein family; it is expressed at very high levels in CD4^+ ^and CD8^+ ^T cells, and in NK cells	SymAtlas
rs7258075	*RYR1*	genic	intron	0.672	Activation of RYR1 causes a rapid increase in the expression of MHCII molecules on the surface of dendritic cells	[57]
rs424138	*DPYSL2*	genic	intron	0.671	Dihydropyrimidinase-like 2	
rs9952350	*ZFP161*	genic	intron, eQTL	0.671	*ZFP161 *encodes a transcriptional repressor expressed at maximum levels in CD4^+ ^T cells	[85], SymAtlas
rs1143683	*ITGAM*	genic	missense (Ala858Val)	0.671	ITGAM combines with ITGB2 to form a leukocyte-specific integrin. ITGAM is the target of an immunomodulatory molecue secreted by *Ancylostoma caninum*	[41]

SNP are ranked according to τ values.

a distance of the associated SNP from the candidate gene. In all cases the candidate gene was the known gene closest to the SNP.

**Figure 1:**
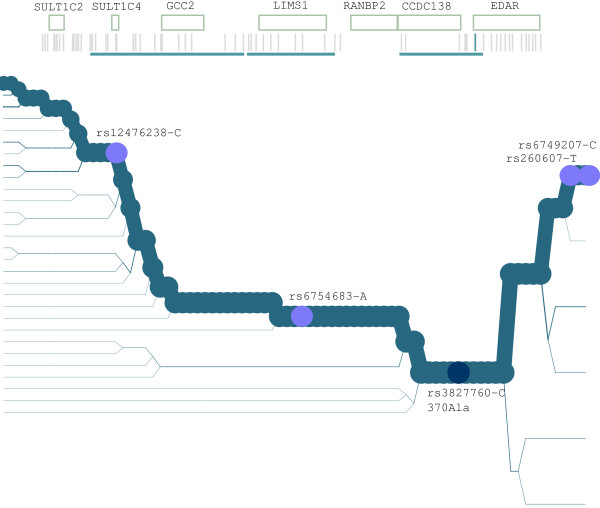
**Haplotype analysis of the genomic region encompassing *EDAR***. Haplotype bifurcation diagram for the genomic region previously identified as being subjected to a selective sweep (see text). The location of known genes in the region is also shown. As described in the text, we selected a core containing the putatively selected 370Val/Ala variant (dark blue circle). The four SNPs that correlate with helminth diversity are represented as light blue circles.

Among the 810 genes subjected to helminth-driven selective pressure, we identified ectodysplasin A (*EDA*) and its receptor (*EDAR*). *EDAR *has been subjected to strong positive selection in populations of Asian descent [17] and is responsible for hair thickness in these populations [18]. One coding variant (rs3827760, 370Val/Ala) in *EDAR *is thought to be the selection target but it has not been genotyped in the HGDP-CEPH panel. We therefore wished to verify whether the variants we found to correlate with helminth diversity are located on the same haplotype as the selected 370Ala allele. We used the Sweep software [19] to analyze the haplotype structure in the genomic region encompassing *EDAR*; we selected a core containing the 370Ala/Val variant and used HapMap data from Asian individuals (Chinese and Japanese). The results indicated that most chromosomes carrying the putatively selected 370Ala variant also harbor four alleles we found to be significantly correlated with helminth diversity (Figure 1).

### Functional characterization of genes subjected to helminth-driven selective pressure

We investigated the role and functional relationship among helminth diversity-associated genes using the Ingenuity Pathway Analysis and the PANTHER classification system [20-22]. For these analyses, a SNP was ascribed to a given gene if it was located within the transcribed region or in the 25 kb upstream of the transcription start site.

**Figure 2:**
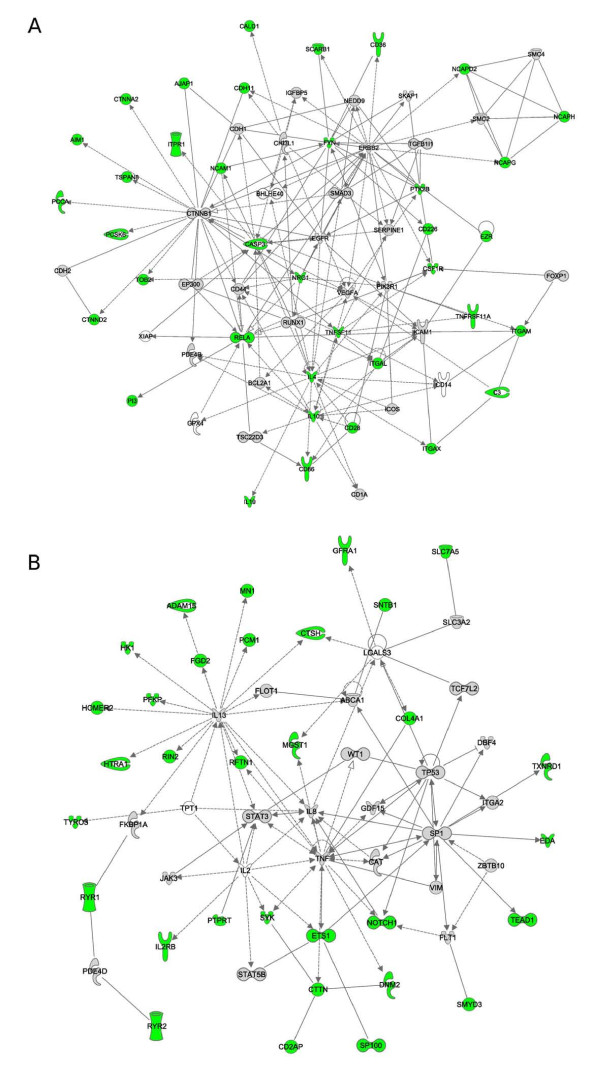
**Network analysis of genes associated with helminth diversity**. Genes are represented as nodes, edges indicate known interactions between proteins (sold lines depicts direct and dashed lines depict indirect interaction). Genes are color coded as follows: green, genes with at least one SNP significantly associated with helminth diversity; gray, genes covered by at least one SNP in the HGDP-CEPH panel; white, genes with no SNPs in the panel. Panel A and B represents the networks obtained by merging networks 1 with 2 and 2 with 5, respectively.

Unsupervised IPA analysis retrieved five high-scoring networks (*p *< 10^-12^) (Figure 2 and Additional file 4, Figure S1) and two additional networks with lower scores (*p *< 10^-9^). The two highest scoring networks were merged, as well as networks 3 and 5. As shown in figure 2, 37 and 32 genes in merged networks 1 and 2 correlated with helminth diversity, respectively, corresponding to almost 60% of network nodes.

We next investigated the over-representation of PANTHER classification categories among genes significantly associated with helminth diversity. Table 3 shows the 5 significantly over-represented PANTHER pathways with the contributing genes, as well as the most significantly over-represented molecular functions and biological processes. Notably, genes involved in cytokine-mediated inflammation and integrin signaling accounted for two significantly over-represented pathways. While the multiple functions of integrins and cytokines on the immune system are established, the role of glutamate and α adrenergic receptor signaling in immune related processes are less understood. Recent evidence has indicated that ionotropic glutamate receptors are expressed by T lymphocytes (reviewed in [23]) and glutamate can exert different actions on these cells including triggering adhesion, proliferation and chemotaxis (reviewed in [23]). Similarly, α adrenergic receptors are expressed by different immunocompetent cells (reviewed in [24]); in particular, expression of *ADRA1A *is induced in human monocytes by inflammatory cytokines [25]. Finally, it is interesting to notice that components of the treleasing hormone (TRH) receptor signaling pathway are also over-represented among genes subjected to helminth driven selective pressure. Tacts on specific receptors within the pituitary gland to stimulate the release of thyroid stimulating hormone and prolactin. The immunomodulatory role of thyroid hormones on immune functions are clearlyestablished (reviewed in [26]). Moreover, experiments in mice have indicated that thyroxine plays a role in the establishment of *Schistosoma mansoni*infection [27,28] and animals treated with the hormone display increased parasite numbers and development of giant worms [28]. Notably, among variants correlating with helminth diversity at the genome-wide level, we also found one SNP relatively close to the gene encoding prolactin (rs13198653, τ = 0.64, *p *= 4.4 × 10^-9^) and one variant within *PRLR *(prolactin receptor, rs4235652, τ = 0.62, *p *= 1.8 × 10^-9^) (Additional file 3, Table S3). PRL was shown to be an immunomodulator and acts as a cytokine on many different immune cells; indeed, *PRLR *is expressed by B, T and NK cells, as well as macrophages (reviewed in [26]) and PRL expression in T cells is regulated by IL-2, IL-4 and IL-1β. These data therefore suggest a role for both thyroid hormones and prolactin in the resistance to parasitic worms.

**Table 3:** Panther over-represented categories among genes showing correlation with helminth diversity

PANTHER category	PANTHER description	Number of genes	p value^a^	Contributing genes
**Pathway**	Integrin signalling pathway	26	0.0067	*ITGAV, COL4A1, ITGA8, FLNB, ITGA9, ITGB8, ITGAM, ITGBL1, COL4A2, COL1A2, DOCK2, PTK2B, ITGAX, FYN, MAPK13, LAMA2, ITGAL, LIMS1, COL24A1, ELMO1, COL15A1, DOCK1, GRB2, ITGB7, MAPK14, COL9A3*
	Alpha adrenergic receptor signaling pathway	8	0.0138	*PLCB1, ADRA1A, ITPR1, GNAQ, SNAP25, PLCE1, VAMP3, ITPR2*
	Inflammation mediated by chemokine and cytokine signaling pathway	31	0.0206	*SOCS6, PLCH2, PLCB1, GRB2, GNG10, ITPR1, CXCR6, MYH14, ITGA9, MYO3B, PLA2G4B, GNAQ, CAMK2A, PLCL1, CAMK2 D, ITGAM, CCR9, COL1A2, ITGB7, PAK7, VAV2, PLCD3, ADCY2, PTK2B, PLCE1, RELA, ITPR2, CCL20, PLA2G4A, ITGAL, COL23A1*
	Ionotropic glutamate receptor pathway	11	0.0263	*SLC17A8, GRIK2, GRIA1, CACNG5, GRIN3A, SHANK2, CAMK2A, SNAP25, CAMK2 D, VAMP3, CACNG8*
	Thyrotropin-releasing hormone receptor signaling pathway	11	0.0303	*PLCH2, PLCB1, CGA, CACNB2, GNG10, GNAQ, PLCD3, CHGA, PLCE1, VAMP3, SNAP25*
**Biological** **process**	Signal transduction	270	4.05 × 10^-17^	n.r.
	Cell adhesion	73	7.18 × 10^-11^	n.r.
	Cell communication	113	9.11 × 10^-10^	n.r.
	Cell structure and motility	103	1.35 × 10^-8^	n.r.
	Neuronal activities	63	2.25 × 10^-8^	n.r.
	Developmental processes	163	2.87 × 10^-8^	n.r.
	Ion transport	64	8.48 × 10^-7^	n.r.
	Cation transport	54	1.61 × 10^-6^	n.r.
**Molecular** **function**	Ion channel	44	4.06 × 10^-7^	n.r.
	Receptor	117	2.79 × 10^-6^	n.r.
	Hydrolase	66	1.92 × 10^-5^	n.r.
	G-protein modulator	45	2.84 × 10^-5^	n.r.
	Cell adhesion molecule	42	3.92 × 10^-5^	n.r.
	Signaling molecule	68	6.44 × 10^-5^	n.r.
	Membrane-bound signaling molecule	20	8.95 × 10^-4^	n.r.

^a ^*p *values are Bonferroni corrected

n.r.: not reported

### Helminth-driven selection and susceptibility to allergy and asthma

We next wished to analyze the relationship between variants/genes associated with helminth diversity and the genetic susceptibility to asthma and allergy. We searched among published genome-wide association studies (GWAS) for SNPs that have been associated with allergy, asthma or related traits (serum IgE levels and plasma eosinophil count). Only 12 SNPs were retrieved, 9 of them genotyped in the HGDP-CEPH panel. One of these SNPs (rs12619285) displayed a significant correlation (*p *= 5.8 × 10^-8^) with helminth diversity (Table 4) and the allele associated with high eosinophil counts [29] positively correlated with the diversity of parasitic worms. In order to gain further insight into this issue, we focused on genes rather than variants, in line with the view that the gene rather than the allele should be regarded as the replication unit. Given the low consistency that often plagues association studies, only robust allergy/asthma susceptibility genes were considered (see methods). As shown in Table 4, we observed that 12 allergy/atopy genes displayed at least one SNP (either genic or intergenic) significantly associated, at the genome-wide level, with helminth diversity. Among these genes, one SNP in *IL4 *that has been associated with asthma (rs2070874, +33C/T) has also been genotyped in the HGDP-CEPH panel; again, the allele that positively correlates with helminth diversity (T) is associated with asthma [30].

**Table 4:** SNPs that correlate with helminth diversity in asthma/allergy genes

SNP	Gene	Distance	τ	Reference
rs2243268 rs2070874	*IL4*	genic genic	0.61 0.60	[9]
rs17316177 rs4368333	*KCNS3*	11291 29279	0.63 0.59	OMIM
rs231735 rs231804 rs11571291	*CTLA4*	38632 23862 11376	0.63 0.60 0.58	[9]
rs4353658 rs7579207	*DPP10*	genic genic	0.62 0.58	[9]
rs1930713 rs2245960 rs7849955	*TLR4*	253946 277604 85563	0.62 0.61 0.59	[9]
rs708491	*PTGER2*	16071	0.58	[9]
rs10905349	*GATA3*	276916	0.57	[9]
rs7329078	*PHF11*	genic	0.57	[9], OMIM
rs1554286	*IL10*	genic	0.57	[9]
rs10237930	*NPSR1*	16676	0.56	[9]
rs877741	*ADRB2*	9418	0.56	[9], OMIM
rs12619285	*IKZF2*	40365	0.56	[29]

**Figure 3:**
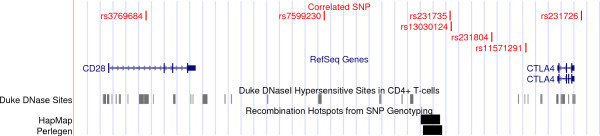
**Analysis of SNP located in the genomic region encompassing *CD28 *and *CTLA4***. SNPs significantly associated with helminth diversity are shown in red while the region covered by *CD28 *and *CTLA4 *are shown in blue. The location of DNAse hypersensitive sites in CD4^+ ^T cells is shown in gray while recombination hotspots are in black. The image was generated by using the "add custom track" utility available through the UCSC Genome Browser.

It is worth noting that we found several SNPs located upstream the transcription start site of *CLTA4 *and subjected to helminth driven selective pressure. *CLTA4 *is located on the long arm of chromosome 2, telomeric to *CD28*. This raises the possibility that the observed allele associations at these two genes (see Figure 3) derive from linkage to a single selected allele. Yet, analysis of linkage disequilibrium (LD) (Additional file 5, Figure S2) indicates that LD is not extensive across the genomic region (also due to the presence of a recombination hot-spot in between the two genes), suggesting that *CD28 *and the promoter region of *CTLA4 *are independent selection targets.

## Discussion

Here we propose that candidate susceptibility genes for parasitic worm infections can be identified by searching for SNPs that display a strong correlation with the diversity of helminth species/genera transmitted in different geographic areas. Our approach relies on the assumption that helminth-driven selective pressure has affected the spatial distribution of these variants and it may suffer from a few limits and caveats. First, we used the diversity of parasitic worms as a measure of helminth-driven selective pressure for the reasons reported above. Yet, helminth diversity may be affected by report biases (e.g. under-reporting in developing countries due to limited research and clinical facilities) and it weights equally all helminth species irrespective of their prevalence and disease burden. As an example, rare helminth species that are reported in Gideon and included in our diversity measure may have exerted a very limited selective force (although prevalence may have changed over time and it is difficult to infer selective pressure for single species). In order to evaluate the impact of rare species, we recalculated helminth diversity by taking into account only parasites that are common in at least one country (see methods and Additional file 1, Table S1). This diversity measure strongly correlated (τ = 0.75, *p *< 10^-5^) with parasite diversity calculated over all species, suggesting that the inclusion of rare helminths should not largely affect our results. Another possible confounding factor is accounted for by the co-variation of helminth diversity with other environmental variables (e.g. climate and other infectious agents). We verified that variants associated with helminth diversity are significantly enriched within immune response genes and that the SNPs we identified do not correlate with climatic factors, suggesting that climate does not act as an important confounding factor. Yet, we cannot rule out the possibility that other infectious agents have affected the spatial distribution of the variants we identified as the diversity of helminths correlates with that of other human pathogens [4,8] across geographic locations.

Finally, although we searched for SNPs strongly associated with helminth diversity (uncorrected *p *value < 7.6 × 10^-8^) and we applied a correction based on the MAF-matched distribution of Kendall's rank correlation coefficients, we cannot exclude that the spatial distribution of a fraction of the variants we identified is due to population demography, migration history and drift, therefore representing false positives.

Despite these limitations we were able to identify several genes that can be regarded as good candidates as modulators of susceptibility to helminth infection as testified by our interaction network and PANTHER analyses. Mammalian hosts respond to parasitic worms in a relatively uniform manner by producing specific cytokines (mainly IL-4, IL-10, IL-5 and IL-13) and IgE, as well as through the activation of effector cells such as eosinophils, basophils and mast cells [31]. Overall, the response to helminth infection is Th2-dominated and serves to both oppose the parasite and to contain tissue-damage. In line with this concept, *IL4 *and *IL10*, as well as *IL13 *are hub genes in two interaction networks we identified (Figure 2). Nonetheless, the role of other immune components during helminth infections is becoming increasingly clear. In addition to Th1 cells that mediate host response in some stages of *Schistosoma *and *Brugia malayi *infection (reviewed in [31,32]), the role of regulatory T cells (Treg) is now recognized (reviewed in [31,32]). An increase in Tregs has been observed in different experimental mouse models of helminthiases and in infected humans (reviewed in [31]). Notably, among the 20 genes more strongly associated with helminth diversity (Table 2) we identified two loci, namely *CD200R1L *[33] and *STIM2 *[34] that are involved in the development and function of Treg cells. Additionally, we found several SNPs located upstream the transcription start site of *CTLA4 *to strongly correlate with helminth diversity (Table 4). The gene encodes a co-inhibitory lymphocyte molecule that is preferentially expressed by Treg cells [35] and is thought to be at least partially responsible for the hypo-responsive phenotype of Th2 effector cells (referred to as "conditioned Th2") which is often observed in helminth infections (reviewed in [31]). As an example, blockade of CTLA-4 during *Nippostrongylus brasiliensis *infection results in higher Th2 cytokine production and decreased parasite numbers [36]. CTLA-4 competes with CD28 for binding to CD86 (and CD80) (reviewed in [37]). Both *CD28 *and *CD86 *carry variants significantly associated with helminth diversity (Figure 3 and Additional file 3, Table S3) and their binding provides a co-stimulatory signal for naive T cells; however, binding of CTLA-4 to CD86/CD80 on dendritic and T cells leads to functional inhibition (reviewed in [37]). Similarly to *CTLA4*, both *CD86 *and *CD28 *have been implicated in the immune response against helminths. Specifically, previous studies have indicated that anti-CD86 treatment blocks immune response to *Schistosoma mansoni *and *Heligmosomoides polygyrus *and *cd28*-/- mice display increased susceptibility to *S. mansoni *[38]. Interestingly, we also found *CD247*, another co-inhibitory molecule to correlate with helminth diversity (Additional file 3, Table S3). A recent report [39] has shown that *Schistosoma *induces anergy of CD4^+ ^and CD8^+ ^T cells by up-regulation of *CD247 *expression on macrophages. These latter cells are considered an important component of anti-helminth response and an alternative form of macrophages has been described in subjects infected by parasitic worms. These cells up-regulate arginase instead of iNOS and express specific molecules including RETNLB (in humans the entry corresponding to *FIZZ1/retnla *has been discontinued and replaced with *RETNLB*) and CHIA (acidic chitinase or AMCase) (reviewed in [31,32]). Notably, we found one SNP in *RETNLB *to significantly correlate with helminth diversity (Additional file 3, Table S3); with respect to *CHIA*, we noticed that one variant (rs10494133) displayed a strong correlation with helminth diversity although it did not withstand Bonferroni correction at the genome-wide level (τ = 0.49, *p *= 3.3 × 10^-6^).

We found several integrins and adhesion molecules to correlate with helminth diversity (Table 3). Among these, *ITGAM*, *ITGAL *and *ITGAX *(Figure 2) encode integrin α chains that combine with the β 2 chain (encoded by *ITGB2*) to form leukocyte-specific heterodimeric integrins. These molecules regulate lymphocyte adhesion and transendothelial migration, playing therefore a central role in inflammatory processes. ITGAL and ITGAM are bound by neutrophil inhibitory factor (NIF), an antiadhesive glycoprotein isolated from the canine hookworm *Ancylostoma caninum *[40,41], suggesting that leukocyte integrins are relevant to the immune response to helminth infections. Interestingly, more recent evidence [42] has revealed that the genome of the human parasite *Necator americanus *encodes at least 9 genes with similarity to NIF, suggesting that their products might play a similar role in establishing an immunocompromised niche for the parasite. The ITGAM/ITGB2 and ITGAX/ITGB2 integrins bind iC3b (Figure 2), a cleavage product of complement component 3 (C3). Previous studies have shown that iC3b is deposited on *N. brasiliensis *larvae [43,44] and *c3 *deficient mice carry high lung larval burdens [43]. In line with these findings, *C3 *is essential for killing *Strongyloides stercoralis *larvae in mice [45] and *c3-/- *mice do not develop an effective Th2 response after infection with *S.mansoni *and cannot clear the parasite after chemotherapy [46].

As far as the second network is concerned (Figure 2), it is worth mentioning that *NOTCH1 *and *ETS1 *have been implicated in multiple immune functions. The NOTCH signaling pathway is involved in the intrathymic differentiation of T cells, as well as in Th cell development in the periphery [47]. In line with the role of Treg cells in helminth infection, *NOTCH1 *has recently been shown to be involved in Treg function [48] and to cooperate with TGFb for regulation of the *FOXP3 *promoter [49]. With respect to *ETS1*, it functions as a transcriptional regulator of several cytokine genes including *IL5*, *IL2 *and *GMCSF *[50-52]. It also regulates expression of *CD226 *[53] and it is known to induce of Th1 mediated inflammation [54].

Also, network B (Figure 2) contains two genes coding for ryanodine receptors (*RYR1 *and *RYR2*); we also found *RYR3 *to correlate significantly with helminth diversity (Additional file 3, Table S3). The function of these molecules in the immune system is poorly understood yet, both *RYR1 *and *RYR3 *have been involved in calcium signaling in T cells [55,56]. Moreover, recent evidences have indicated that dendritic cells express *RYR1 *and activation of the receptor causes a rapid increase in the expression of MHCII molecules on the surface of these cells [57].

Finally, it is interesting to notice that among the genes subjected to helminth-driven selective pressure in network B we found *SYK*, encoding a tyrosine kinase that interacts with the high affinity IgE receptor and mediates IgE signaling in mast cells and basophils [58,59]. Similarly, *CD226 *and *FYN *(both in network A, Figure 2) have been involved in mast cell activation mediated by the high affinity IgE receptor [60], suggesting a role for these genes in allergic inflammation. Indeed, *syk *has been shown to mediate airway hyper-responsiveness in an experimental mouse model [61] and most genes discussed above have been involved in the elicitation of allergic phenomena. In addition genes reported in Table 4, the interaction of CD28 with CD86 is central to induction of allergic airway inflammation in mice [62] and CD86 antisense oligonucleotides suppress airway hyper-responsiveness in allergic animals [63]. Variants in C3 have been associated with asthma [64] and mice deficient in C3 exhibit diminished airway hyper-responsiveness and lung eosinophilia when challenged with allergen [65]; also, NOTCH1 is involved CD8^+ ^T cell-mediated development of airway hyper-responsiveness and inflammation [66], while ITGAL/ITGB2 mediates altered responsiveness of atopic asthmatic airway smooth muscle in rabbits [67]. Finally, Ets-1 induces tenascin expression in bronchial fibroblasts [68]. In this respect it is worth mentioning that, although subjects genotyped in the HGDP-CEPH panel are supposed to be healthy, a proportion of them may suffer from relatively mild diseases including asthma, atopy and related disorders; this may be especially true in some areas such as Latin America, for example, where urban centres have the highest reported prevalence of asthma worldwide (reviewed in [69]). While this possibility does not affect the results we reported herein, it highlights the fact that the epidemiology of these disorders is rapidly changing, and several reports have revealed a general increase in prevalence with urbanization, leading to the suggestion that environmental factors (including helminth infections) may play a central role in modulating the susceptibility to these diseases (reviewed in [69]). The relationship between asthma/allergy susceptibility and parasitic worms is though to be complex (reviewed in [70]). On one hand helminth-driven selective pressure is expected to favor individuals carrying alleles that allow a strong Th2 response and, therefore to promote the transmission and spread of asthma-susceptibility variants. On the other hand, lack of parasites in developed countries has likely removed the immunomodulatory role of these organisms, eventually leading to the increased incidence of atopic conditions. The current knowledge of asthma/allergy susceptibility alleles (12 alleles identified by GWAS) is too limited to warrant extensive speculation on the first issue. Still, our data indicate that many genes we identified carry variants associated with asthma/allergy or have been involved in the elicitation of airway hyper-responsiveness. Therefore, our results expand the previously noticed parallelism between genes involved in the development of asthma/allergy and those responsible for responding to parasitic worms, suggesting that the evolutionary scenario underlying the increase in asthma, allergy and related phenotype envisages a relevant role for these long-standing parasites.

Among the genes subjected to helminth-driven selective pressure we identified *EDAR *and *EDA*, its ligand. Binding of ectodysplasin to EDAR activates the NF-kB pathway through the NEMO protein. The EDA/EDAR pair mediates signals needed for the development of ectodermal appendages and mutations in both genes result in hypohidrotic ectodermal dysplasia. Many studies [15,17,71,72] have indicated that *EDAR *has been subjected to a strong selective pressure resulting in the rapid spread of the putatively selected 370Ala allele in Asian populations. This allele is responsible for the hair phenotype of these populations but the selective pressure underlying the selective sweep is unknown. Hypotheses have been proposed that increased hair thickness might be protective against cold climates or be favored through sexual selection [18,73]. We found that in Asian populations, most chromosomes carrying the selected allele also carry four SNPs subjected to helminth-driven selective pressure (Figure 1). Both *EDA *and *EDAR *are expressed in human lymphocytes and dendritic cells (see methods), suggesting that they may function as NF-kB activators in these cell types, as well. It is therefore tempting to speculate that helminths represent the selective pressure underlying the spread of a selected allele in Asia. This idea is consistent with the concept whereby infectious agents have represented one of the major selective forces for human populations.

## Conclusions

In summary, our data are consistent with the notion whereby parasitic worms have acted as a powerful selective force on human populations and have contributed to shape nucleotide variability at a number of genes involved in immune responses. We also show that several genes associated with helminth diversity are involved in the pathogenesis of atopic conditions or in airway hyper-responsiveness.

## Methods

### Data retrieval and statistical analysis

Helminth absence/presence matrices for the 21 countries where HGDP-CEPH populations are located were derived from the Gideon database. Information in Gideon is weekly updated and derives from World Health Organization reports, National Health Ministries, PubMed searches and epidemiology meetings. The Gideon Epidemiology module follows the status of known infectious diseases globally, as well as in individual countries, with specific notes indicating the disease's history, incidence and distribution per country. We manually curated helminth absence/presence matrices by extracting information from single Gideon entries. Following previous suggestions [4-6], we recorded only helminths that are transmitted in the 21 countries, meaning that cases of transmission due to tourism and immigration were not taken into account. A total of 60 helminth species were identified in at least one country (Additional file 6, Table S4). Prevalence data for single helminth infections were similarly obtained from Gideon, as described in the text. In order to calculate parasite diversity for species that are common in at least one country, we inspected Gideon entries for survey data or prevalence notes; helminth infections reported as "rare in humans" were discarded; similarly, parasites with no prevalence estimates or notes were considered as rare; therefore, this diversity measure should be regarded as an approximate estimate.

The annual minimum and maximum temperature were retrieved from the NCEP/NCAR database (Legates and Willmott Average, re-gridded dataset) using the geographic coordinates reported by HGDP-CEPH website for each population. Similarly, net short wave radiation flux data were obtained from NCEP/NCAR (Reanalysis 1: Surface Flux); these data were read using Grid Analysis and Display System (GrADS).

Since helminth diversity, due to data organization in Gideon, can only be calculated per country (rather than per population), the same procedure was applied to climatic variables. Therefore the values of annual temperature, radiation flux and precipitation rate were averaged for populations located in the same country. This assures that a similar number of ties is maintained in all correlation analyses.

Data concerning the HGDP-CEPH panel derive from a previous work [11]. Atypical or duplicated samples and pairs of close relatives were removed [74]. Following previous indications [4,5], Bantu individuals (South Africa) were considered as one population.

A SNP was ascribed to a specific gene if it was located within the transcribed region or no farther than 500 bp upstream the transcription start site. MAF for any single SNP was calculated as the average over all populations. The list of immune response genes was derived from the Immunology Database and Analysis Portal (ImmPort). Expression data were obtained from SymAtlas. SNPs identified in GWAS and associated with allergy, asthma or related traits (serum IgE levels and plasma eosinophil count) were derived from the A Catalog of Published Genome-Wide Association Studies. The list of allergy/asthma susceptibility genes was obtained from a previous review [9] or from the Online Medelian Inheritance in Man website (MIM: 600807).

All correlations were calculated by Kendall's rank correlation coefficient (τ), a non-parametric statistic used to measure the degree of correspondence between two rankings. The reason for using this test is that even in the presence of ties, the sampling distribution of τ satisfactorily converges to a normal distribution for values of *n *larger than 10 [75].

In order to estimate the probability of obtaining 246 genes carrying at least one significantly associated SNP out of a group of 2,287 genes (the number of ImmPort genes), we applied a re-sampling approach after dividing genes on the basis of the number of SNPs typed in the HGDP-CEPH panel. In particular, all genes covered by at least one SNP in the HGDP-CEPH panel (number of genes = 15,280) were divided in 24 intervals based on the distribution of typed SNPs per gene (Additional file 7, Table S5). Samples of 2,287 genes were randomly extracted from a list of all genes covered by at least one SNP in the HGDP-CEPH panel by applying the criterion that for each ImmPort gene, a control gene was selected from the same interval. For each sample the number of genes with at least one significant SNP were counted. The empirical probability of obtaining 246 genes was then calculated from the distribution of counts deriving from 10,000 random samples. Similarly, the number of SNPs in ImmPort genes was compared to the distribution of SNPs in the 10,000 re-samplings.

Analysis of PANTHER over-represented functional categories and pathways was performed using the "Compare Classifications of Lists" tool available at the PANTHER classification system website. Briefly, gene lists are compared to the reference list using the binomial test [22] for each molecular function, biological process, or pathway term in PANTHER. All *p *values were Bonferroni corrected. All calculation were performed in the R environment [76]. For PANTHER analysis we widened the inclusion criteria in that SNPs located within the transcribed region or in the 25 kb upstream the transcription start site were ascribed to the gene.

eQTL data were derived from the eQTL Resource web site held at the University of Chicago (Prtichard Lab).

### Network construction

Biological network analysis was performed with Ingenuity Pathways Analysis (IPA) software using an unsupervised analysis. IPA builds networks by querying the Ingenuity Pathways Knowledge Base for interactions between the identified genes and all other gene objects stored in the knowledge base; it then generates networks with a maximum network size of 35 genes/proteins. We used all genes showing at least one significantly associated SNP as the input set; in this case a SNP was ascribed to a gene if it was located within the transcribed region or in the 25 kb upstream. All network edges are supported by at least one published reference or from canonical information stored in the Ingenuity Pathways Knowledge Base. To determine the probability of the analyzed genes to be found together in a network from Ingenuity Pathways Knowledge Base due to random chance alone, IPA applies a Fisher's exact test. The network score represents the -log (*p *value).

## Abbreviations

SNP: single nucleotide polymorphism; Treg: regulatory T cell; LD: linkage disequilibrium; MAF: minor allele frequency; NIF: neutrophil inhibitory factor; TRH: treleasing hormone; PRL: prolactin.

## Authors' contributions

MF, UP, RC and MS performed the analyzes, analyzed and interpreted the data; GPC and NB participated in the study coordination; MC and MS conceived the study and wrote the paper.

All authors read and approved the final manuscript.

## LINKS

The Immunology Database and Analysis Portal, https://www.immport.org

Ingenuity Pathway Analysis, Ingenuity Systems, http://www.ingenuity.com

NCEP/NCAR, Surface flux, http://www.esrl.noaa.gov/psd/data/gridded/data.ncep.reanalysis.surfaceflux.html

GrADS, http://www.iges.org/grads/

SymAtlas,: http://symatlas.gnf.org

Catalog of published GWAS, http://www.genome.gov

Panther,: http://www.pantherdb.org

eQTL resources @ the pritchard lab, http://eqtl.uchicago.edu/Home.html

UCSC Genome Browser, http://genome.ucsc.edu

HGDP-CEPH Panel, http://hagsc.org/hgdp/

Sweep software, http://www.broadinstitute.org/mpg/sweep/

## Supplementary Material

Additional file 1 **Table S1**. Populations in the HGDP-CEPH panel and helminth diversity estimates. Click here for file

Additional file 2 **Table S2**. Genes in the ImmPort list that display at least one SNP significantly associated with helminth diversity. For each gene the SNP showing the strongest correlation is reported. SNPs are ranked according to the value of τ. Click here for file

Additional file 3 **Table S3**. SNPs significantly associated with helminth diversity. The table reports all SNPs that withstood Bonferroni correction at the genome-wide level (with alpha = 0.5) and displayed a tau percentile rank higher than the 95th among MAF-matched SNPs, as described in the main text. SNPs are ranked according to the value of τ. If the SNP is located within a genic region (or in the 500 upstream nucleotides) the gene symbol is reported. Alternatively, the gene closest to the SNP and its distance (in bp) are indicated. Click here for file

Additional file 4 **Figure S1**. In addition to the two merged networks in the main text, IPA identified three additional networks (A-C) with *p *< 10^-9^. Genes are represented as nodes, edges indicate known interactions between proteins (sold lines depicts direct and dashed lines depict indirect interaction). Genes are color coded as follows: green, genes with at least one SNP significantly associated with helminth diversity; gray, genes covered by at least one SNP in the HGDP-CEPH panel; white, genes with no SNPs in the panel. Click here for file

Additional file 5 **Figure S2**. Analysis of LD in the genomic region encompassing *CD28 *and *CTLA4*. SNPs significantly associated with helminth diversity are shown in red while the region covered by *CD28 *and *CTLA4 *are shown in blue. The location of DNAse hypersensitive sites in CD4^+ ^T cells is shown in gray while recombination hot-spots are in black. LD plots (r2) are shown for Yoruba (YRI), Europeans (CEU) and Asians (JPT+CHB). The image was generated by using the "add custom track" utility available through the UCSC Genome Browser. Click here for file

Additional file 6 **Table S4**. Helminth species/genera transmitted in at least one country and that are common in at least one country. Click here for file

Additional file 7 **Table S5**. Gene subdivision on the basis of SNP number. Genes were divided in 24 intervals according to the number of SNPs typed in the HGDP-CEPH panel. Click here for file
