# Supplementary figures and images for: The landscape of human genes involved in the immune response to parasitic worms
Source: BMC Evol Biol. 2010 Aug 31;10:264. doi: 10.1186/1471-2148-10-264 (PMC2940816; doi:10.1186/1471-2148-10-264)

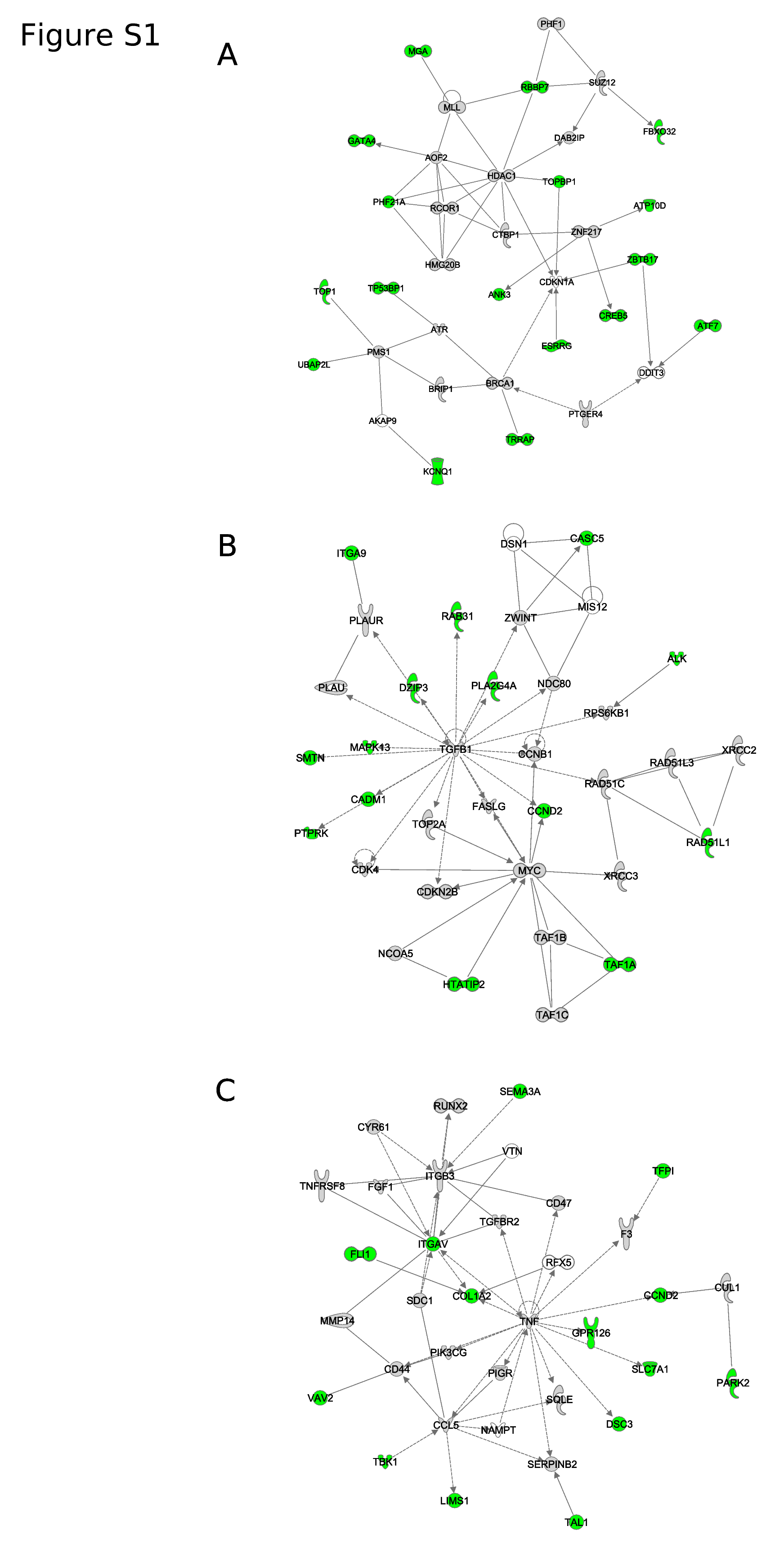

Supplement: Additional file 4 — Figure S1. In addition to the two merged networks in the main text, IPA identified three additional networks (A-C) with p < 10-9. Genes are represented as nodes, edges indicate known interactions between proteins (sold lines depicts direct and dashed lines depict indirect interaction). Genes are color coded as follows: green, genes with at least one SNP significantly associated with helminth diversity; gray, genes covered by at least one SNP in the HGDP-CEPH panel; white, genes with no SNPs in the panel. [file 1471-2148-10-264-S4.TIFF]

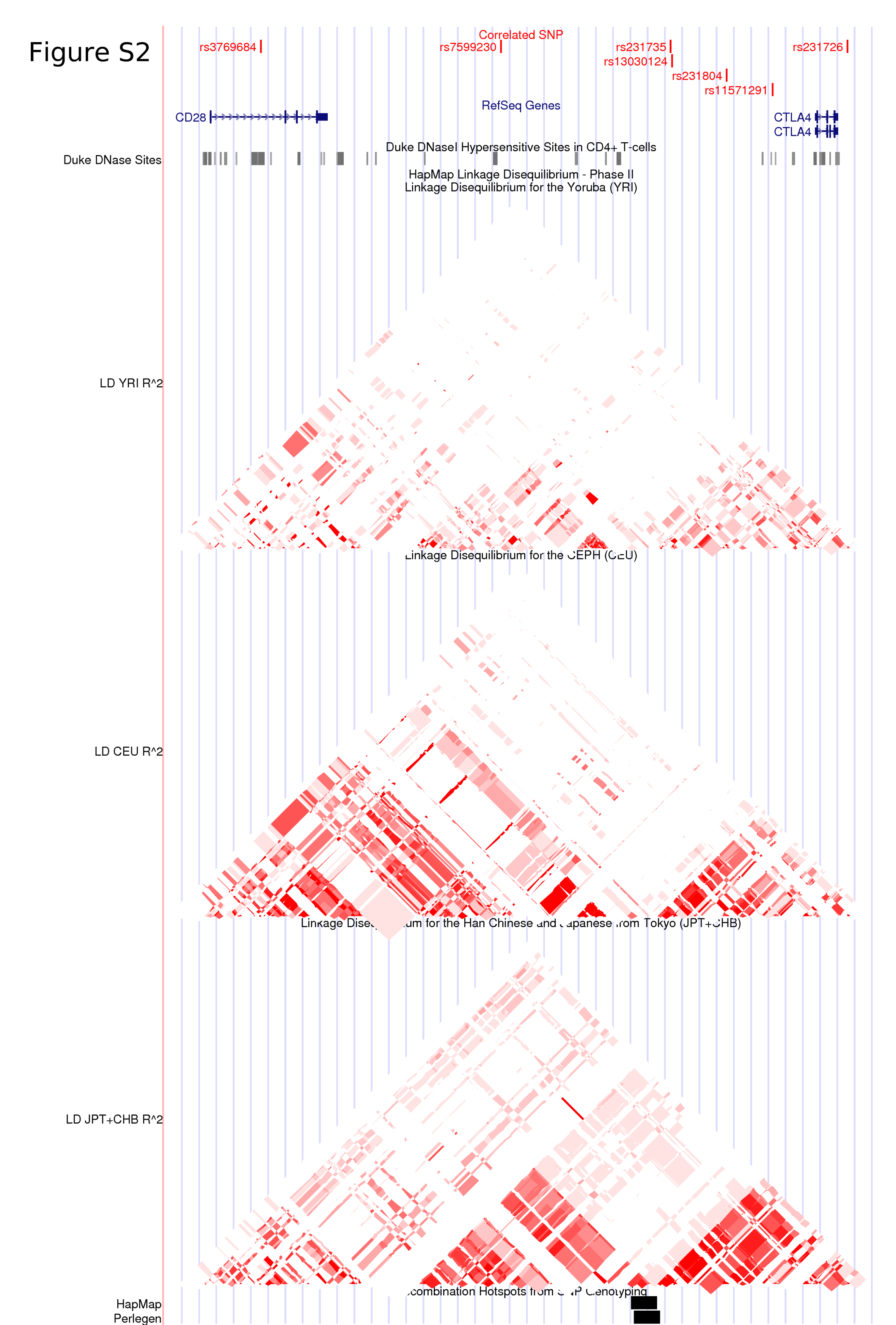

Supplement: Additional file 5 — Figure S2. Analysis of LD in the genomic region encompassing CD28 and CTLA4. SNPs significantly associated with helminth diversity are shown in red while the region covered by CD28 and CTLA4 are shown in blue. The location of DNAse hypersensitive sites in CD4+ T cells is shown in gray while recombination hot-spots are in black. LD plots (r2) are shown for Yoruba (YRI), Europeans (CEU) and Asians (JPT+CHB). The image was generated by using the "add custom track" utility available through the UCSC Genome Browser. [file 1471-2148-10-264-S5.TIFF]
