# Supplementary material for: The landscape of human genes involved in the immune response to parasitic worms
Source: BMC Evol Biol. 2010 Aug 31;10:264. doi: 10.1186/1471-2148-10-264 (PMC2940816; doi:10.1186/1471-2148-10-264)
Supplement: Additional file 6 — Table S4. Helminth species/genera transmitted in at least one country and that are common in at least one country. [file 1471-2148-10-264-S6.DOC]

**Table S4.**

Helminth species/genera transmitted in at least one country

| Helminths (60) |
| --- |
| *Angiostrongylus cantonensis*, *Prastrongylus costaricensis*, *Necator americanus, Capillaria hepatica* and *C. aerophila*, *Capillaria philippinensis*, *Dioctophyme renalis*, *Dracunculus medinensis*, *Wuchereria bancrofti*, *Brugia malayi*, *Gnathostoma spinigerum*, *Gongylonema pulchrum*, *Lagochilascaris minor*, *Loa loa*, *Mammomonogamus laryngeus*, *Mansonella ozzardi*, *Mansonella perstans*, *Mansonella streptocerca*, *Oesophagostomum bifurcum*, *Onchocerca volvulus*, *Trichostrongylus axei* or *T. brevis* or *T. capricola* or *T. colubriformis* or *T. lerouxi or T. orientalis* or *T. vitrinus,* *Anisakis simplex*, *Pseudoterranova decipiens, Ascaris lumbricoides*, *Bunostomum phlebotomum*, *Strongyloides myopotami*, *Dirofilaria immitis*, *Dirofilaria tenuis*, *Dirofilaria ripens*, *Dirofilaria ursi*, *Enterobius vermicularis*, *Ancylostoma duodenale*, *Strongyloides stercoralis*, *Thelazia callipaeda*, *Toxocara cati* and *canis*, *Trichinella spiralis*, *Trichuris trichiura*, *Bertiella studeri* or *Bertiella mucronata*, *Inermicapsifer arvicanthidid* or *I. madagascariensis* or *I. cubensis, Avian schistosomes* (*Trichobilharzia*, *Heterobilharzia*, *Orientobilharzia*), *Clonorchis sinensis*, *Taenia multiceps*, *Dicrocoelium dendriticum* and *D. hospes*, *Echinococcus vogeli* and *E. oligarthrus*, *Echinococcus multilocularis*, *Echinostomatida* (*Echinostoma ilocanum*, *E. malayanum*, *E. revolutum*), *Fasciola hepatica* and *Fasciola gigantica*, *Fasciolopsis buski*, *Gastrodiscoides hominis, Heterophyes heterophyes*, *Metagonimus yokogawai*, *Metorchis conjunctus* and *M. orientalis*, *Nanophyetus salmincola*, *Opisthorchis felenius* or *O. guayaquilensis* or *O. viverrini*, *Paragonimus westermani* or *P. heterotemus* or *P. skrjabini* or *P. miyazakii* or *P. africanus*, *Schistosoma haematobium*, *Schistosoma intercalatum*, *Schistosoma mansoni*, *Schistosoma japonicum*, *Schistosoma mattheei*, *Schistosoma mekongi*, *Spirometra mansoni* or *S. mansonoides* or *S. theileri* |

Helminth species/genera that are common in at least one country (and are not transmitted in all countries)

| Helminths (28) |
| --- |
| *Angiostrongylus cantonensis*, *Prastrongylus costaricensis*, *Necator americanus, Capillaria philippinensis*, *Dracunculus medinensis*, *Wuchereria bancrofti*, *Brugia malayi*, *Gnathostoma spinigerum*, *Loa loa*, *Mansonella ozzardi*, *Oesophagostomum bifurcum*, *Onchocerca volvulus*, *Anisakis simplex*, *Clonorchis sinensis*, *Echinococcus multilocularis*, *Echinostomatida* (*Echinostoma ilocanum*, *E. malayanum*, *E. revolutum*), *Fasciola hepatica* and *Fasciola gigantica*, *Fasciolopsis buski*, *Heterophyes heterophyes*, *Metagonimus yokogawai*, *Opisthorchis felenius* or *O. guayaquilensis* or *O. viverrini*, *Paragonimus westermani* or *P. heterotemus* or *P. skrjabini* or *P. miyazakii* or *P. africanus*, *Schistosoma haematobium*, *Schistosoma intercalatum*, *Schistosoma mansoni*, *Schistosoma japonicum*, *Schistosoma mattheei*, *Schistosoma mekongi*, *Spirometra mansoni* or *S. mansonoides* or *S. theileri* |
