# Supplementary material for: The landscape of human genes involved in the immune response to parasitic worms
Source: BMC Evol Biol. 2010 Aug 31;10:264. doi: 10.1186/1471-2148-10-264 (PMC2940816; doi:10.1186/1471-2148-10-264)
Supplement: Additional file 7 — Table S5. Gene subdivision on the basis of SNP number. Genes were divided in 24 intervals according to the number of SNPs typed in the HGDP-CEPH panel. [file 1471-2148-10-264-S7.PDF]

Table S5. Gene subdivision on the basis of SNP number. Genes were divided in 24 intervals according to the number of SNPs typed in the HGDP-CEPH panel.

Percentiles	SNP number	Number of ImmPort genes	Number of non-ImmPort genes
[0,20)	[1,2)	564	3526
[20,40)	[2,3)	319	2135
[40,60)	[3,8)	390	2414
[60,80)	[8,18)	431	2444
[80,81	[18,19)	24	110
[81,82)	[19,20)	22	107
[82,83)	[20,21)	19	85
[83,84)	[21,23)	49	180
[84,85)	[23,24)	12	85
[85,86)	[24,26)	37	135
[86,87)	[26,28)	32	109
[87,88)	[28,30)	29	117
[88,89)	[30,33)	29	147
[89,90)	[33,36)	32	114
[90,91)	[36,40)	25	131
[91,92)	[40,45)	38	144
[92,93)	[45,49)	9	100
[93,94)	[49,57)	33	144
[94,95)	[57,67)	30	121
[95,96)	[67,81)	33	125
[96,97)	[81,99)	31	131
[97,99)	[99,124)	29	131
[98,99)	[124,182)	23	128
[99,100]	[182,1857)	47	130
